# Size matters: Sample size assessments for chronic wasting disease surveillance using an agent-based modeling framework

**DOI:** 10.1016/j.mex.2020.100953

**Published:** 2020-06-11

**Authors:** Aniruddha Belsare, Matthew Gompper, Barbara Keller, Jason Sumners, Lonnie Hansen, Joshua Millspaugh

**Affiliations:** a Boone & Crockett Quantitative Wildlife Center, Michigan State University, East Lansing, MI 48824, United States; b Department of Fish, Wildlife and Conservation Ecology, New Mexico State University, Las Cruces, NM 88003, United States; c Minnesota Department of Natural Resources, St. Paul, MN 55155, United States; d Missouri Department of Conservation, 3500 East Gans Road, Columbia, MO 65201, United States; e W.A. Franke College of Forestry and Conservation, University of Montana, Missoula, MT 59812, United States

**Keywords:** Agent-based modeling, Harvest-based surveillance, NetLogo, Iterative model analysis

## Abstract

Epidemiological surveillance for many important wildlife diseases relies on samples obtained from hunter-harvested animals. Statistical methods used to calculate sample size requirements assume that the target population is randomly sampled, and therefore the samples are representative of the population. But hunter-harvested samples may not be representative of the population due to disease distribution heterogeneities (e.g. spatial clustering of infected individuals), and harvest-related non-random processes like regulations, hunter selectivity, variable land access, and uneven hunter distribution. Consequently, sample sizes necessary for detection of disease are underestimated and disease detection probabilities are overestimated, resulting in erroneous inferences about disease presence and distribution.

We have developed a modeling framework to support the design of efficient disease surveillance programs for wildlife populations. The constituent agent-based models can incorporate real-world heterogeneities associated with disease distribution, harvest, and harvest-based sampling, and can be used to determine population-specific sample sizes necessary for prompt detection of important wildlife diseases like chronic wasting disease and bovine tuberculosis. The modeling framework and its application has been described in detail by Belsare et al. [1]. Here we describe how model scenarios were developed and implemented, and how model outputs were analyzed. The main objectives of this methods paper are to provide users the opportunity to a) assess the reproducibility of the published model results, b) gain an in-depth understanding of model analysis, and c) facilitate adaptation of this modeling framework to other regions and other wildlife disease systems.

• The two agent-based models, MO*Ov*POP and MO*Ov*POP*surveillance*, incorporate real-world heterogeneities underpinned by host characteristics, disease spread dynamics, and sampling biases in hunter-harvested deer.
• The modeling framework facilitates iterative analysis of locally relevant disease surveillance scenarios, thereby facilitating sample size calculations for prompt and reliable detection of important wildlife diseases.
• Insights gained from modeling studies can be used to inform the design of effective wildlife disease surveillance strategies.

## Graphical abstract

**Figure:**
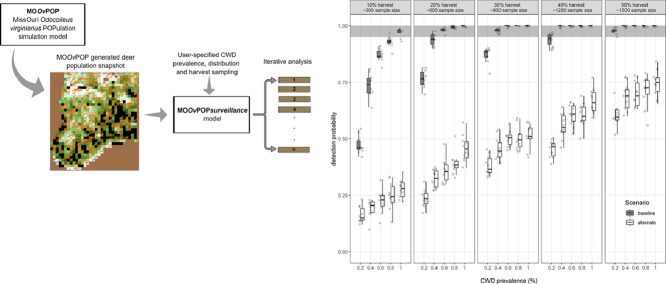

Specifications Table

**Table:** 

Subject Area	Agricultural and Biological Sciences
More specific subject area	*Wildlife disease surveillance, agent-based simulation modeling*
Method name	Iterative analysis using an agent-based modeling framework
Name and reference of original method	*Not applicable*
Resource availability	*Model code, data (GIS files, population snapshots), documentation, and model output files (for Franklin County; analysis described in this paper) are all available for download here: MOOvPOP*https://www.comses.net/codebases/5585/releases/2.2.0/
	*MOOvPOPsurveillance*https://www.comses.net/codebases/5576/releases/2.2.0/
	*R code files for analysis of model output data, with links to relevant model output files in a Github repository, are available here:*https://github.com/anyadoc/FranklinCWDsurveillance_Rcode

## Method details

Surveillance for important wildlife diseases often relies on samples obtained from hunter-harvested animals. But sampling biases associated with harvest and spatiotemporal heterogeneities in disease distribution may result in biased estimates and erroneous inferences about disease presence and distribution. Yet, harvest-based sampling is widely used by wildlife agencies as it is a convenient and cost-effective mechanism of obtaining wildlife samples. Chronic wasting disease (CWD) surveillance of wild cervid populations in North America is a case in point.

CWD is an emerging prion disease of cervids (including white-tailed deer *Odocoileus virginianus*, mule deer *Odocoileus hemionus*, and elk *Cervus elaphus*), and its continuing spread poses a serious long-term threat to the health of free-ranging cervid populations. In many states and provinces across North America, wildlife agencies obtain samples from harvested deer for CWD surveillance programs. We have developed an agent-based modeling framework that can be used as a decision-support tool for designing efficient harvest-based CWD surveillance strategies. The constituent models of this framework incorporate real-world heterogeneities in disease distribution, hunter harvest and harvest-based sampling, and can be used to determine population-specific sample sizes for reliable and prompt detection of the disease.

The modeling framework was developed for, and in collaboration with, wildlife agency biologists and managers. Two agent-based models, MO*Ov*POP (MissOuri *Odocoileus virginianus* POPulation simulation model) and MO*Ov*POP*surveillance*, constitute the framework. Both models are coded in NetLogo, an open source Java-based modeling environment, and model programs are freely available in the digital repository CoMSES Net Computational Model Library [2, 3]. Model programs developed using NetLogo are user friendly primarily due to the graphical user interface (GUI). The interface sliders and choices allow users (even non-modelers) to update model assumptions based on their current best knowledge of the system and perform virtual experiments.

We have described the application of this modeling framework in the context of CWD surveillance in Missouri [1]. Here, we describe the methods used for model evaluation and application (or how the models can be used to support CWD surveillance). Specifically, we describe how model scenarios were designed and implemented. The data and analysis presented in this article pertain to model versions 2.2.0 (both models) simulated for Franklin County, Missouri. Data files, model documents and instructions required to run the model are downloaded along with the model codes.

User-specified information (landscape, vital rates, harvest rates, disease prevalence and distribution) underpins model simulations. MO*Ov*POP simulates a realistic deer population in a user-generated landscape and MO*Ov*POP*surveillance* uses a snapshot of the *in silico* deer population to simulate disease prevalence and distribution, harvest effort and sampling.

Forest cover data is used to simulate the distribution of deer in MO*Ov*POP landscape. We have converted the forest cover data (United States Geological Survey 1992 National Land Cover Data) to a forest percentage grid of one square mile patches for select counties in Missouri (downloaded along with the model code in data folder). But MO*Ov*POP can be setup using GIS coverage data (forest cover) for any region of interest. Step-by-step instructions for incorporating new landscapes in MO*Ov*POP:

1. Convert the forest cover data for the region of interest to a forest percentage grid of one square mile patches. Save this using the ASCII grid file format (.asc). Note the ncols and nrows (highlighted in Fig. 1).

**Fig. 1:**
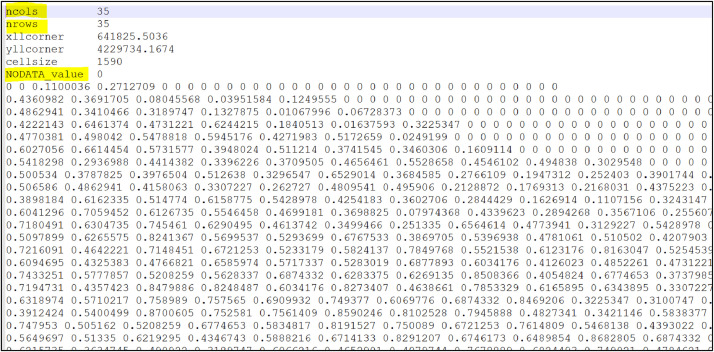
A snapshot of the GIS data (forest cover) for Franklin County Missouri stored using the ASCII file format (.asc). Note the values for ‘ncols’ and ‘nrows’, and change ‘NODATA_value’ from 0 to −9999 (highlighted).
2. Change the NODATA_value from 0 to −9999 (highlighted in Fig. 1). Save the changed file in the data folder.
3. Click the ‘Code’ tab on MO*Ov*POP interface. Click the ‘Procedures’ tab and select ‘setup-landscape’. This will bring up the part in the program where you can add the new landscape to the model. The code snippet for adding Franklin County in the model is provided as an example. Ensure that correct resize-world values are entered (ncols – 1 and nrows – 1).
4. On the MO*Ov*POP interface, right click on the ‘region’ tab and select ‘Edit’. Type the name of the new region here as shown in Fig. 2.

**Fig. 2:**
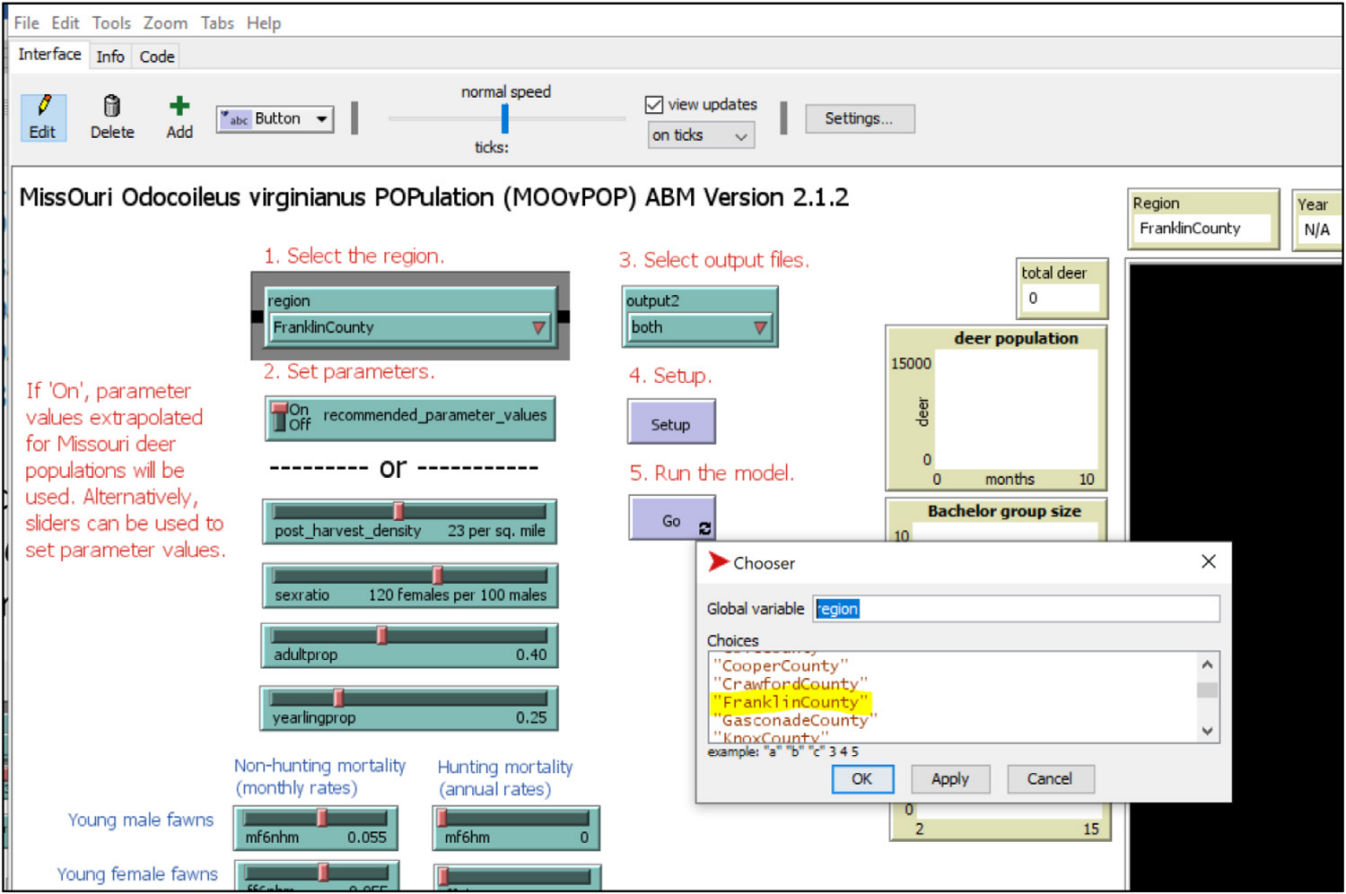
The procedure for adding new region to MO*Ov*POP is illustrated on the model's Graphical User Interface (GUI).
5. Click the ‘File’ tab and save the changes.

## Running MO*Ov*POP

Three types of NetLogo interface widgets are provided on the Graphical User Interface to set user-specified parameters: sliders, choosers and a switch (Fig. 2). Parameters are specified before running the model. Parameter values derived for Franklin County deer population are provided in Table 1. First, click the ‘Setup’ button. Once the setup is completed (Setup button changes back to blue color), click the ‘Go’ button to start a model run. The model runs for 25 years, and for every year of the model run, population and harvest data are documented in an output file ‘deerpopdy*CountyName*.csv’ (e.g. deerpopdyFranklinCounty.csv). Another output file ‘saRegion.csv’ (e.g. saFranklinCounty.csv) is also written in the results folder. This file documents the abundance, age class proportion and female: male ratio for every year of model run. The output files are saved in the ‘results’ folder.

**Table 1:** Deer population parameter values for simulating Franklin County deer population using MO*Ov*POP. Parameter values are derived from field-based surveys and harvest data collected by the Missouri Department of Conservation (MDC).

Parameter	Description	Value
*post_harvest_density*	Initial deer density (per forested sq. mile)	23
*sexratio*	Male: female ratio in the population	1:1.2
*adultprop*	Adult proportion (≥ 25 months) in the population	0.4
*yearlingprop*	Yearling proportion in the population	0.25

## MO*Ov*POP evaluation

**Fig. 3:**
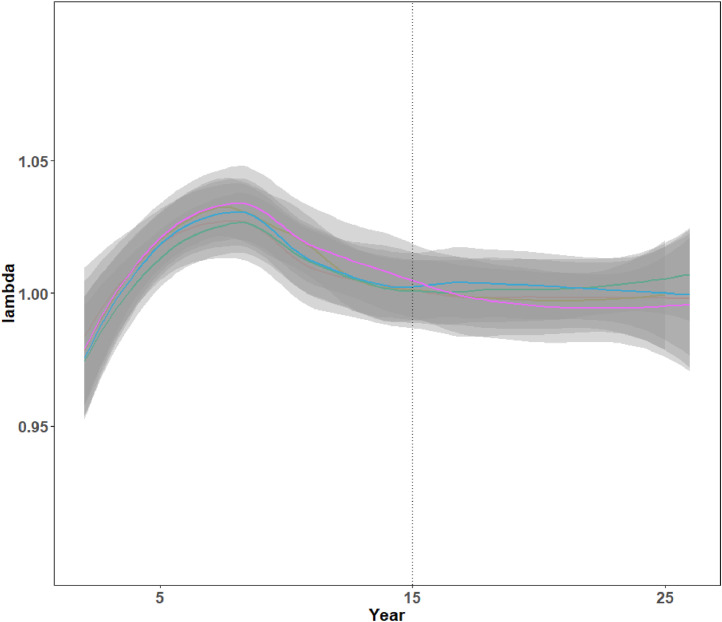
Finite population growth rate (λ) for the five MO*Ov*POP generated Franklin County deer populations. Each line represents one model iteration.

**Fig. 4:**
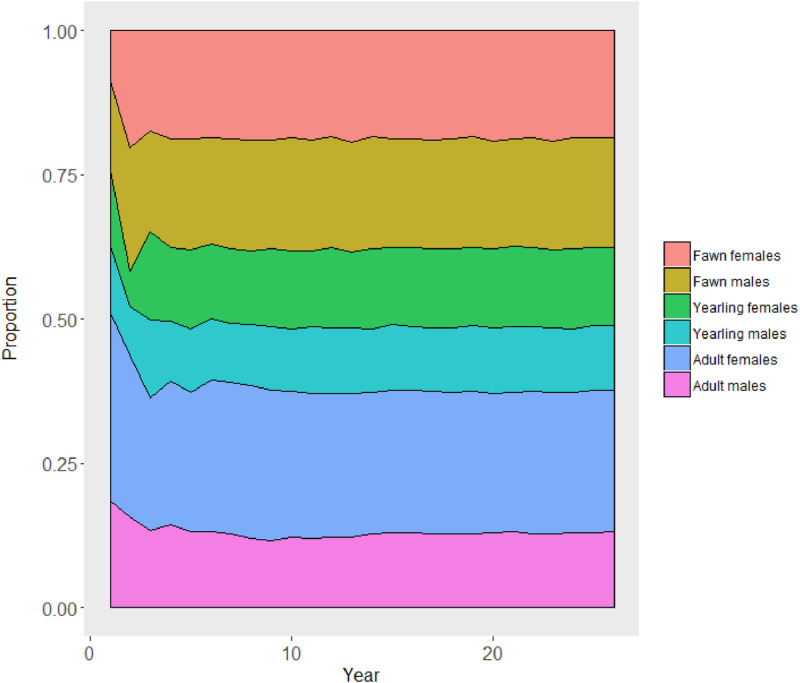
Age-sex composition of MO*Ov*POP simulated Franklin County deer population over a period of 25 years (one model iteration).

MO*Ov*POP is designed to generate a realistic *in silico* deer population that can be used to initialize other agent-based models (surveillance model or CWD dynamics model). We used data from five MO*Ov*POP iterations (output file *deerpopdyFranklinCounty.csv*) to assess finite population growth rate (lambda) and age structure of the model-generated deer populations. Pre-harvest abundance from year two onwards was used to calculate lambda (Fig. 3). Age-sex composition of the population was assessed using post-harvest abundance for each year of the model run (one iteration) (Fig. 4).

We recommend using BehaviorSpace (a software tool integrated with NetLogo, accessed using the ‘Tools’ dropdown menu) when multiple iterations are to be executed.

Output file for the five MO*Ov*POP iterations is also available here:

https://github.com/anyadoc/FranklinCWDsurveillance_Rcode/blob/master/deerpopdyFranklinCounty_5iterations.csv

**Fig. 5:**
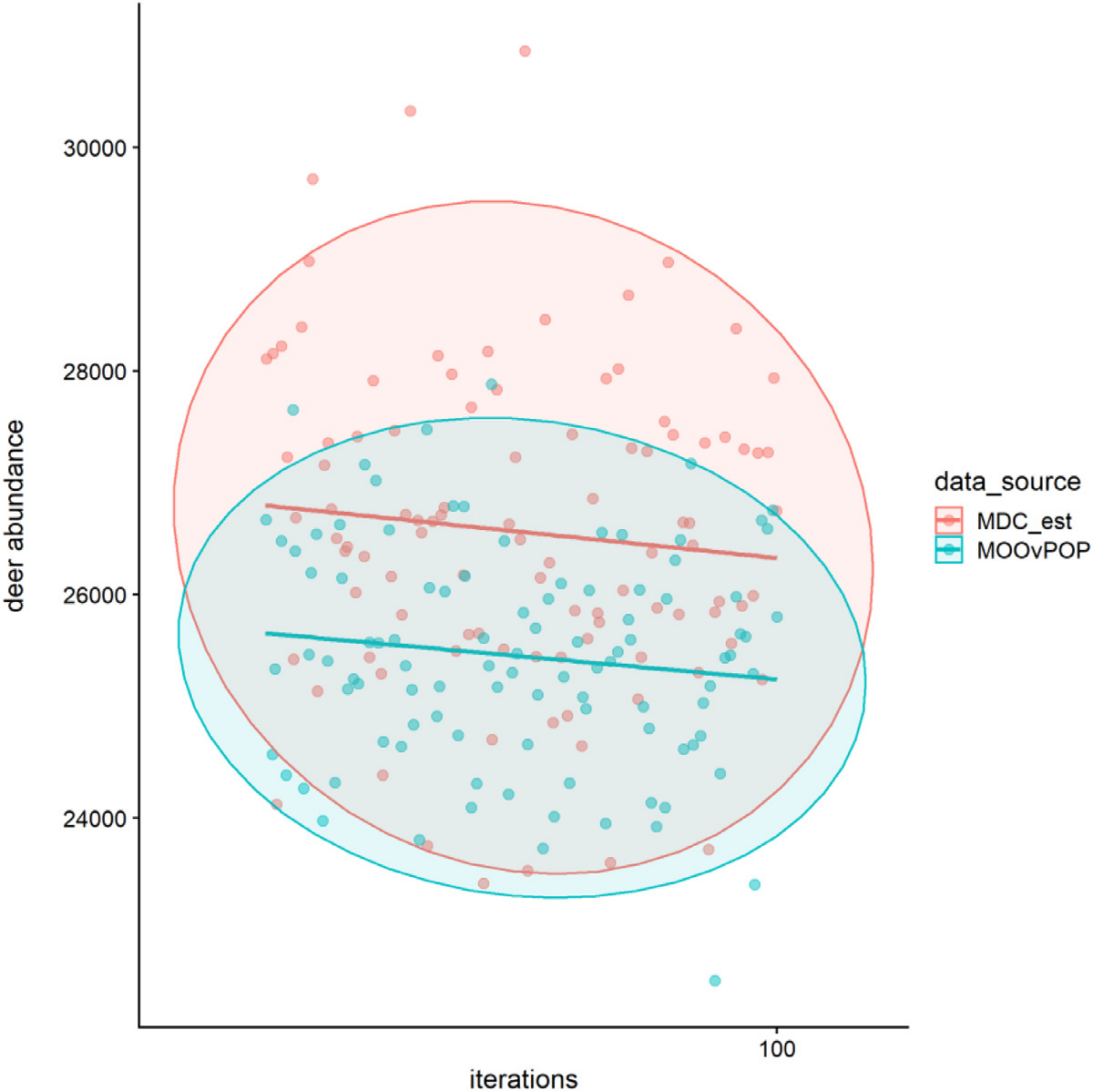
Plot comparing pre-harvest deer abundance in MO*Ov*POP generated populations (26th year snapshots from 100 iterations) with Missouri Department of Conservation's estimate (MDC_est: 26,502 ± 5%) for Franklin County, Missouri.

We then completed 100 MO*Ov*POP iterations and analyzed the 26th year population snapshots to assess the congruence of model-generated populations with field estimates for Franklin County deer population (abundance, age structure and sex ratio). The model output file ‘saFranklinCounty.csv’ can be used to compare the pre-harvest model deer abundance (26th year) with Missouri Department of Conservation's (MDC) estimate of 26,502 for year 2016 (Fig. 5). We assume a standard deviation of 5% for MDC's abundance estimate.

Output file for the 100 MO*Ov*POP iterations is also available here: https://github.com/anyadoc/FranklinCWDsurveillance_Rcode/blob/master/saFranklinCounty_100.csv

R code to generate Figs. 3, 4 and 5 with links to the relevant model output files is available here:

https://github.com/anyadoc/FranklinCWDsurveillance_Rcode/blob/master/MOOvPOPanalysis_Rcode.R

This file includes code to summarize age class proportion and sex ratio in the model generated deer populations.

## MO*Ov*POP*surveillance* evaluation

MO*Ov*POP*surveillance* allows the user to simulate hypothetical CWD prevalence and distribution pattern, and then implements harvest-based sampling to test for CWD. We use model iterations to determine the effects of alternate assumptions (or scenarios) on CWD detection probability. Specifically, the model is iterated 100 times and the proportion of iterations where at least one CWD+ deer is detected is the detection probability. We use two scenarios to simulate CWD distribution in the deer population and the nature of sampling process.

a) Baseline scenario: CWD+ deer were randomly distributed in the deer population. Hunter harvest was also simulated as a random process. On the GUI, Chooser cwd_dist was set to ‘random_dist’, and Chooser sampling to ‘random_sampling’.
b) Alternate scenario: CWD+ deer were clustered (100% cases on <5% landscape). Hunter harvest was simulated as a non-random process (15% deer habitat patches were set as high harvest patches where 50% of the total harvest occurs). On the GUI, Chooser cwd_dist was set to ‘clustered_dist’ and Chooser sampling to ‘non-random sampling’.

We further simulated four prevalence-sample size combinations under the baseline scenario to evaluate MO*Ov*POP*surveillance* performance. Only the adult male component of Franklin County deer population was considered for these simulations. The adult male abundance in the MO*Ov*POP derived pre-harvest snapshot is ~4170 (check the last row (for year 26) in column J ('preh_ma') in the output file ‘deerpopdyFranklinCounty.csv’).

1. Using the total number of adult male deer in this population (4170), we determine number of infected deer for each prevalence scenario: 0.5% prevalence ~ (4170 * 0.5) / 100 = 20.85 ~ 21; 1% prevalence ~ 42; 2% prevalence ~ 83 and 5% prevalence ~ 209.
2. Only males were considered for these evaluations, hence the slider ‘*m-f-prevalence*’ was set to 1.
3. The total adult population is 10,384: adult males (last row column J) 4170 + adult females (last row column M) 6214. If 0.5% prevalence is to be simulated, 21 adult males out of 10,384 adults would have to be designated as CWD+ (Note that only adult males will be selected as we have set the slider ‘*m-f-prevalence*’ at 1). Therefore, the ‘*adult-prevalence*’ slider should be set at 0.002 (21 / 10,384 = 0.002). In the same way, we calculated the ‘*adult-prevalence*’ slider settings for other prevalence scenarios (Table 3). Sliders ‘*fawn-prevalence*’ and ‘*yearling-prevalence*’ are set to 0.
4. We then determined sample sizes using hypergeometric approximation for the four prevalence levels. Standardized sample size tables and online calculators like EpiTools (Sergeant, ESG, 2018. Epitools epidemiological calculators. Ausvet Pty Ltd. Available at: http://epitools.ausvet.com.au) are available for calculating sample sizes. Using the population size 4170 (can be approximated to 4500 or 4000 if using sample size tables), we determined sample sizes for 90%, 95% and 99% detection probability for each prevalence scenario.
5. To set the ‘*% adult male harvest tested*’ slider, we first determined the total male adult harvest (which is 1629 - from the last row of column R of ‘deerpopdyFranklinCounty.csv’) and then divided the sample size calculated for each scenario by this number. We further calibrated ‘*% adult male harvest tested*’ iteratively to ensure that number of samples tested match with the calculated sample size (run the model and check output file 'CWDsurveillanceMO.csv' column R). Calibrated slider settings for all MO*Ov*POP*surveillance* scenarios are provided in Table 2.

**Table 2:** Calibrated settings for MO*Ov*POP*surveillance* evaluation scenarios. These scenarios are simulated using baseline assumptions, i.e. random distribution of CWD+ individuals and random sampling.

Prevalence scenario	Confidence level	Sample size	*%adult-male-harvest-tested*	*m-f-prev-ratio*	*adult-prevalence*
0.5%	0.90	433	0.255	1	0.002
0.5%	0.95	554	0.325	1	0.002
0.5%	0.99	820	0.48	1	0.002
1%	0.90	222	0.13	1	0.004
1%	0.95	287	0.17	1	0.004
1%	0.99	432	0.255	1	0.004
2%	0.90	113	0.065	1	0.008
2%	0.95	147	0.085	1	0.008
2%	0.99	223	0.13	1	0.008
5%	0.90	46	0.025	1	0.02
5%	0.95	59	0.035	1	0.02
5%	0.99	90	0.055	1	0.02
6. For each scenario, detection probability was determined from 1000 iterations of MO*Ov*POP*surveillance* (10 replicates of 100 iterations). Using a single sample *t*-test, we determined if statistically significant difference existed between the model-derived and hypergeometric distribution derived detection probabilities.

Output files for MO*Ov*POP*surveillance* evaluation scenarios are also available here: https://github.com/anyadoc/FranklinCWDsurveillance_Rcode (CWDsurveillianceMO_sc1.csv to CWD surveillianceMO_sc12.csv).

## Sensitivity analysis: MO*Ov*POP*surveillance*

**Fig. 6:**
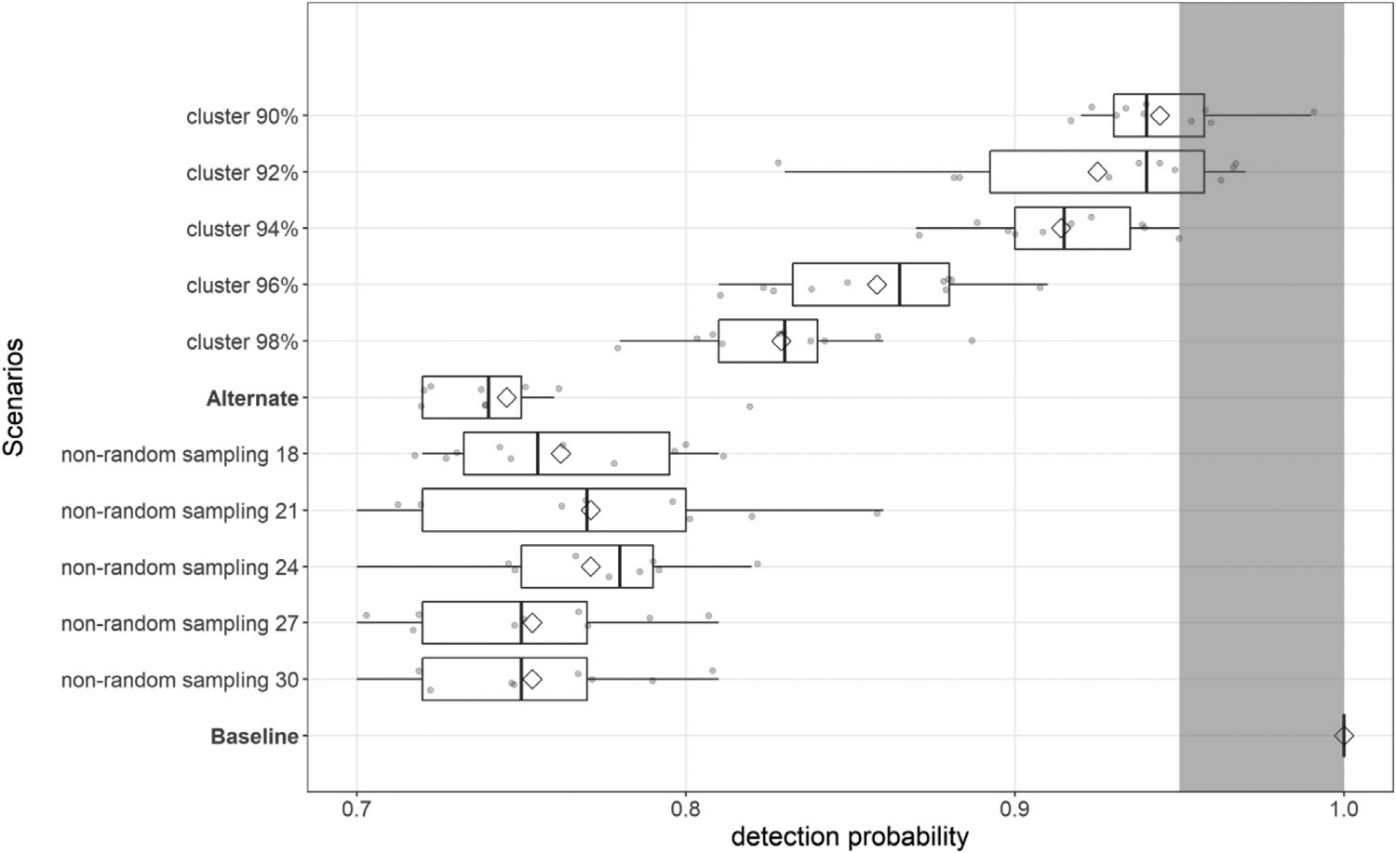
Plot comparing model-derived detection probabilities for sensitivity analysis scenarios.

We explored how sensitive model output (detection probability) was to changes in a) how disease clustering was simulated, and b) how non-random sampling was simulated (Fig. 6). We set the prevalence in adult deer at 1% (‘adult-prevalence’ slider set to 0.01), and sample size at 50% of the adult harvest (both ‘%adult-male-harvest-tested’ and ‘%adult-female-harvest-tested’ sliders set to 0.5). We assessed the sensitivity of model derived CWD detection probability using the following scenarios (10 replicates of 100 MO*Ov*POP*surveillance* iterations for each scenario):

1. Baseline
2. Alternate
  Rest of clustering and sampling patterns use Alternate scenario settings. For scenarios 3 to 7: Click on the ‘code’ tab of MO*Ov*POPsurveillance and comment out lines 174 to 181, as well as lines 815 to 822. Commenting out is done by adding a semicolon at the beginning of a code line.
3. cluster98%: 2% cases distributed outside the cluster. Lines 816 and 819 change to 0.98, lines 818 and 821 change to 0.02.
4. cluster96%: 4% cases distributed outside the cluster. Lines 816 and 819 change to 0.96, lines 818 and 821 change to 0.04.
5. cluster94%: 6% cases distributed outside the cluster. Lines 816 and 819 change to 0.94, lines 818 and 821 change to 0.06.
6. cluster92%: 8% cases distributed outside the cluster. Lines 816 and 819 change to 0.92, lines 818 and 821 change to 0.08.
7. cluster90%: 10% cases distributed outside the cluster. Lines 816 and 819 change to 0.90, lines 818 and 821 change to 0.1.
  For scenarios 8 to 12: Comment out lines 174 to 181, as well as lines 815 to 822.
8. nrs18: 18% of deer habitat patches are set as high harvest pressure patches where 50% of the total harvest occurs. Change value in line 216 to 0.18.
9. nrs21: 21% of deer habitat patches are set as high harvest pressure patches where 50% of the total harvest occurs. Change value in line 216 to 0.21.
10. nrs24: 24% of deer habitat patches are set as high harvest pressure patches where 50% of the total harvest occurs. Change value in line 216 to 0.24.
11. nrs27: 27% of deer habitat patches are set as high harvest pressure patches where 50% of the total harvest occurs. Change value in line 216 to 0.27.
12. nrs30: 30% of deer habitat patches are set as high harvest pressure patches where 50% of the total harvest occurs. Change value in line 216 to 0.30.

Output files for MO*Ov*POP*surveillance* sensitivity analysis scenarios (CWDsurveillanceMO_bl1000.csv, CWDsurveillanceMO_alt1000.csv, nrs18 to nrs30, cl901000 to cl981000) available here: https://github.com/anyadoc/FranklinCWDsurveillance_Rcode

R code for a) MO*Ov*POPsurveillance evaluation (to calculate detection probabilities for 12 prevalence-sample size scenarios and compare each with detection probability derived from hypergeometric approximation using a one-sample *t*-test), and b) sensitivity analysis of MO*Ov*POPsurveillance and Fig. 6, with links to relevant model output files, is available here: https://github.com/anyadoc/FranklinCWDsurveillance_Rcode/blob/master/MOOvPOPsurvEvalSA_Rcode.R

## Model application

**Fig. 7:**
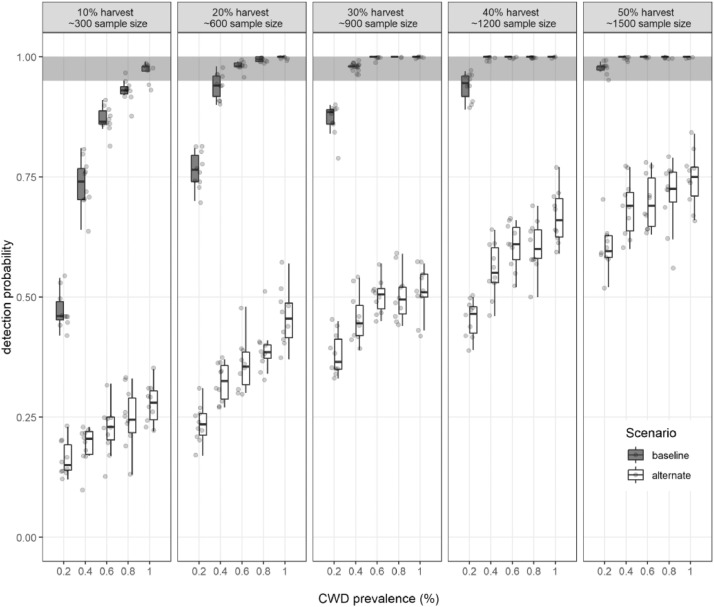
Plot showing the detection probabilities derived from iterative analysis of 25 baseline and 25 alternate scenarios. Each circle represents detection probability determined from 100 model iterations.

MO*Ov*POP*surveillance* was used to determine sample sizes that have high detection probabilities for hypothesized population prevalence rates using different assumptions (random CWD distribution, random sampling, clustered CWD distribution, non-random sampling). We simulated a range of prevalence-sample size combinations for Franklin County deer population using baseline and alternate scenarios (as described under sensitivity analysis). Specifically, we tested five prevalence levels in adult deer (0.2%, 0.4%, 0.6%, 0.8% and 1%). Sample sizes were simulated as percent of the total adult deer harvested (10%, 20%, 30%, 40% and 50%). The adult deer harvest in our model (~3000) approximates the adult harvest during 2016 Fall Firearm harvest in Franklin County (MDC Deer Harvest Summary 2016–2017). Slider settings for both baseline and alternate scenarios simulated using MO*Ov*POPsurveillance are provided in Table 3. Detection probabilities estimated from 10 replicates of 100 model iterations for each prevalence-sample size scenario are presented in Fig. 7.

**Table 3:** Graphical User Interface settings for MO*Ov*POP*surveillance* baseline and alternate scenarios (Model Application).

Scenario (baseline/alternate)	*adult-prevalence*	*m-f-prev-ratio*	*%adult-male-harvest-tested*	*%adult-female-harvest-tested*
1	0.002	0.5	0.1	0.1
2	0.002	0.5	0.2	0.2
3	0.002	0.5	0.3	0.3
4	0.002	0.5	0.4	0.4
5	0.002	0.5	0.5	0.5
6	0.004	0.5	0.1	0.1
7	0.004	0.5	0.2	0.2
8	0.004	0.5	0.3	0.3
9	0.004	0.5	0.4	0.4
10	0.004	0.5	0.5	0.5
11	0.006	0.5	0.1	0.1
12	0.006	0.5	0.2	0.2
13	0.006	0.5	0.3	0.3
14	0.006	0.5	0.4	0.4
15	0.006	0.5	0.5	0.5
16	0.008	0.5	0.1	0.1
17	0.008	0.5	0.2	0.2
18	0.008	0.5	0.3	0.3
19	0.008	0.5	0.4	0.4
20	0.008	0.5	0.5	0.5
21	0.01	0.5	0.1	0.1
22	0.01	0.5	0.2	0.2
23	0.01	0.5	0.3	0.3
24	0.01	0.5	0.4	0.4
25	0.01	0.5	0.5	0.5

Output files for MO*Ov*POP*surveillance* model application scenarios are also available here: https://github.com/anyadoc/FranklinCWDsurveillance_Rcode (MO*Ov*POP*surveillance*: model application scenarios bl1 to bl25 and alt1 to alt25).

R code for analyzing MO*Ov*POP*surveillance* application scenario outputs and to generate the graph in Fig. 7, with links to relevant model output files, is available here: https://github.com/anyadoc/FranklinCWDsurveillance_Rcode/blob/master/MOOvPOPsurvApp_Rcode.R

## Declaration of Competing Interests

The authors declare that they have no known competing financial interests or personal relationships that could have appeared to influence the work reported in this paper.

## References

[1] Belsare A., Gompper M., Keller B., Sumners J., Hansen L., Millspaugh J. (2020). An agent-based framework for improving wildlife disease surveillance: a case study of chronic wasting disease in Missouri white-tailed deer. Ecol. Modell..

[2] A. Belsare, M. Gompper, J. Millspaugh, (2020). MO*Ov*POP (Version 2.2.0). CoMSES Computational Model Library. Retrieved from: https://www.comses.net/codebases/5585/releases/2.2.0/.

[3] A. Belsare, M. Gompper, J. Millspaugh, (2020). MO*Ov*POPsurveillance. (Version 2.2.0). CoMSES Computational Model Library. Retrieved from: https://www.comses.net/codebases/5576/releases/2.2.0/.

